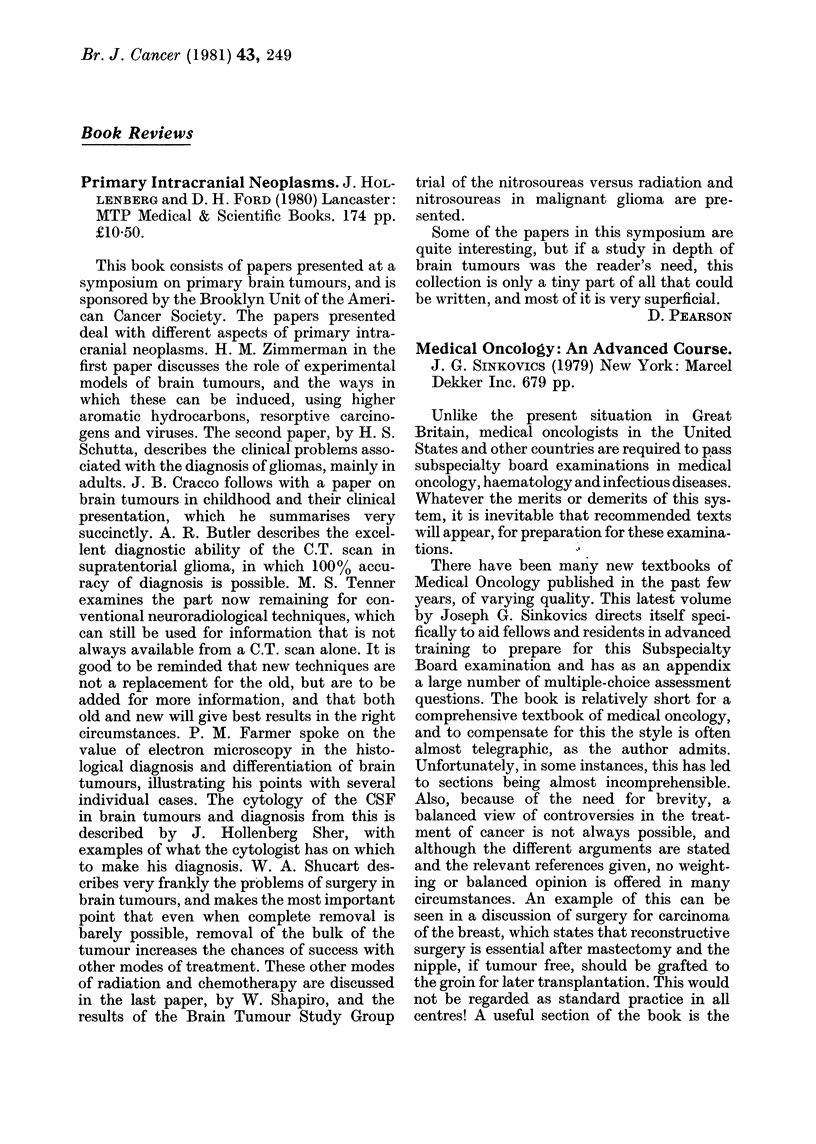# Primary Intracranial Neoplasms

**Published:** 1981-02

**Authors:** D. Pearson


*Br. J. Cancer* (1981) **43**, 249

### *Book Reviews*

**Primary Intracranial Neoplasms.** J. Hollenberg and D. H. Ford (1980) Lancaster: MTP Medical & Scientific Books. 174 pp. £10·50.

This book consists of papers presented at a symposium on primary brain tumours, and is sponsored by the Brooklyn Unit of the American Cancer Society. The papers presented deal with different aspects of primary intracranial neoplasms. H. M. Zimmerman in the first paper discusses the role of experimental models of brain tumours, and the ways in which these can be induced, using higher aromatic hydrocarbons, resorptive carcinogens and viruses. The second paper, by H. S. Schutta, describes the clinical problems associated with the diagnosis of gliomas, mainly in adults. J. B. Cracco follows with a paper on brain tumours in childhood and their clinical presentation, which he summarises very succinctly. A. R. Butler describes the excellent diagnostic ability of the C.T. scan in supratentorial glioma, in which 100% accuracy of diagnosis is possible. M. S. Tenner examines the part now remaining for conventional neuroradiological techniques, which can still be used for information that is not always available from a C.T. scan alone. It is good to be reminded that new techniques are not a replacement for the old, but are to be added for more information, and that both old and new will give best results in the right circumstances. P. M. Farmer spoke on the value of electron microscopy in the histological diagnosis and differentiation of brain tumours, illustrating his points with several individual cases. The cytology of the CSF in brain tumours and diagnosis from this is described by J. Hollenberg Sher, with examples of what the cytologist has on which to make his diagnosis. W. A. Shucart describes very frankly the problems of surgery in brain tumours, and makes the most important point that even when complete removal is barely possible, removal of the bulk of the tumour increases the chances of success with other modes of treatment. These other modes of radiation and chemotherapy are discussed in the last paper, by W. Shapiro, and the results of the Brain Tumour Study Group trial of the nitrosoureas versus radiation and nitrosoureas in malignant glioma are presented.

Some of the papers in this symposium are quite interesting, but if a study in depth of brain tumours was the reader's need, this collection is only a tiny part of all that could be written, and most of it is very superficial.

D. Pearson